# Urban cultural heritage is mentally restorative: an experimental study based on multiple psychophysiological measures

**DOI:** 10.3389/fpsyg.2023.1132052

**Published:** 2023-05-17

**Authors:** ShuSheng Wang, Yuqian Xu, Xinyi Yang, Yuan Zhang, Pei Yan, Yuan Jiang, Kai Wang

**Affiliations:** ^1^State Key Laboratory of Green Building in Western China, Xi'an University of Architecture and Technology, Xi'an, China; ^2^College of Architecture, Xi'an University of Architecture and Technology, Xi'an, China; ^3^School of Psychology, Shaanxi Normal University, Xi'an, China; ^4^China Academy of Urban-Rural Development and Cultural Heritage, Xi'an, China

**Keywords:** historical building, cultural heritage, electrophysiology response, eye movement, fascination, restorative

## Abstract

**Introduction:**

Urban cultural heritage sites bear the cultural functions of a city, hold spiritual and cultural value, can recall emotional memories, and serve the cultural leisure activities of the residents. Urban cultural heritage sites can help citizens perceive a sense of belonging and a feeling of relaxation, but whether and to what extent cultural heritage sites affect mental health remains unknown.

**Methods:**

Based on attention restoration theory, multiple research methods are adopted in this study to examine the impact of cultural heritage on human restorative mechanisms. Five representative cultural heritage sites from the cultural heritage-rich city of Xi'an are selected as the research object. In addition, a questionnaire survey and physiological experiments are conducted. Perceived restorative scale, skin conductance response, heart rate variability, and eye movement data while viewing photographs of the cases are collected from the participants.

**Results:**

Results show that cultural heritage sites have psychophysiological restorative effects, which are especially significant in the fascination dimension. Moreover, historical buildings can promote the restorative effects of cultural heritage sites.

**Discussion:**

This finding may lead to new conservation and innovation planning strategies considering the mental health effects of cultural heritage.

## 1. Introduction

Urban cultural heritage is an important part of a city, which bears its cultural functions, holds spiritual and cultural value, recalls residents' emotional memories, and serves their cultural leisure activities (Wang, 2018). Accordingly, previous studies extensively investigated and interpreted urban cultural heritage sites in terms of their assessment (Sowinska-Swierkosz, 2017), esthetic value (Yoshimura and Hiura, 2017), perceived affective quality, and restorative potential (Scopelliti et al., 2019). Urban cultural heritage may not only give a city a soul but also help citizens perceive a sense of belonging and a feeling of relaxation. Substantial records exist of the ability of cultural heritage to “clear one's mind and get rid of anxiety” (Gao et al., 2020) in ancient China. For instance, a visit to the Wang Min Pavilion, which was constructed in the Song Dynasty, with a view of the Min Mountain, was claimed to improve one's eyesight and clear one's mind (Zhang, Song Dynasty). Meanwhile, Pi Yun Tower (He, Ming Dynasty) and Qing Xin Pavilion (Wu, Qing Dynasty) could also induce such kinds of feelings. These records reveal the potential restorative value of urban cultural heritage sites.

Facing the high stress, density, noise, and pollution levels of their cities, growing evidence suggests that residents are vulnerable to mental problems. To address the issues regarding this problem, numerous studies focused on the mental health benefits of green spaces (Mitchell, 2013; Carrus et al., 2017; Wood et al., 2018; Collins et al., 2020; de Bell et al., 2020). However, other types of urban spaces are necessary, which may be conducive to mental health (Browning et al., 2019). In addition, other types of spaces that can help people relax must be found or created. How space can generate a feeling of relaxation should also be determined.

The aforementioned discussion is based on attention restoration theory (Kaplan and Kaplan, 1989), which proposes individuals' attention and mental status recovery by accessing the natural environment (Kaplan, 1995), and stress reduction theory (Ulrich et al., 1991), which states that decreased stress and increased positive emotions from the natural environment can induce restorative effects (Ulrich et al., 1991). Scholars also proved that certain types of urban spaces (Negrin et al., 2017), except natural space, can be restorative, such as urban green space (Jiang et al., 2014, 2016; Elyssa et al., 2016), public space (César et al., 2017; Rios-Rodriguez et al., 2021), historical areas (Scopelliti et al., 2019), streets (Lin et al., 2014; Bornioli et al., 2018), cemeteries (Nordh et al., 2017), and some indoor environments (Yin et al., 2020). Previous studies also found that architectural characteristics may be more significant than vegetation to restoration (Lindal and Hartig, 2015). Various architectural variations and low heights can lead to restoration; for example, the rich façade of historical buildings was observed to be restorative (Galindo and Hidalgo, 2005; Fornara and Troffa, 2009). Moreover, the effects of architectural elements on restoration were reflected in the dimensions of fascination and being away (Kaplan, 1995). Meanwhile, other studies reported that historical areas are highly relevant in terms of the fascination dimension of restoration (Scopelliti et al., 2019).

An urban cultural heritage site is a special type of space consisting of green space and historical buildings, with considerable esthetic value (Yoshimura and Hiura, 2017) and non-material benefits and may be conducive to mental health. Urban cultural heritage sites are recognized as spaces that provide valuable cultural ecosystem services or have historical value and may improve mental health and physical wellbeing (Van Berkel et al., 2018; Cervera et al., 2021). Some psychological experiences brought by cultural spaces on mental health might be relevant to cultural belonging, place attachment (Liu et al., 2020), and esthetic preference (Purcell et al., 2001; Galindo and Hidalgo, 2005; Hoyle et al., 2017; Tieskens et al., 2022). As for Chinese landscape preference, the dominant factors affecting it involved progressive enhancement with tranquility, diversity, traditional characteristics, and maintenance of buildings and human constructions (Ren, 2019). Some scholars showed the importance of historical monuments and attractions in addition to specific environmental features that are appreciated by the public (Van Berkel et al., 2018). If people lack awareness of the landscape, there will be certain obstacles to obtaining the potential for health and wellbeing (Cervera et al., 2021). All of these have proved the potential restorative value of cultural heritage. However, their effects on residents' mental health and restorative mechanism are underexplored. Therefore, research on the effect of cultural heritage on mental health is critical and urgent.

The aforementioned theories and studies emphasized the potential of cultural heritage sites to restore mental states, which deserves further investigation. In this study, we aimed to address this issue, as such knowledge may be helpful to society as a guide on how to improve citizens' mental health. However, cultural heritage may have value for mental health but not only in terms of cultural inheritance. Thus, in this study, we ask the following questions: “What is the extent of the restorative effects of cultural heritage sites?,” “What is the difference between green spaces and cultural heritage sites in terms of restoration, and what are the reasons behind this difference?,” and “What are the similarities and differences between each cultural heritage site?” To answer these questions, we conduct a survey and experiments using multiple psychophysiological measures to assess the restorative effects of cultural heritage sites.

## 2. Methods

This section is divided into three parts. The first part is the Materials section, where we select five sites; the second part is the Measures section, where we introduce the multiple measures, including the questionnaire and physiological responses; and the third part is the Procedures section, where we describe the pilot study and quasi-experimental design.

### 2.1. Materials

**Figure 1:**
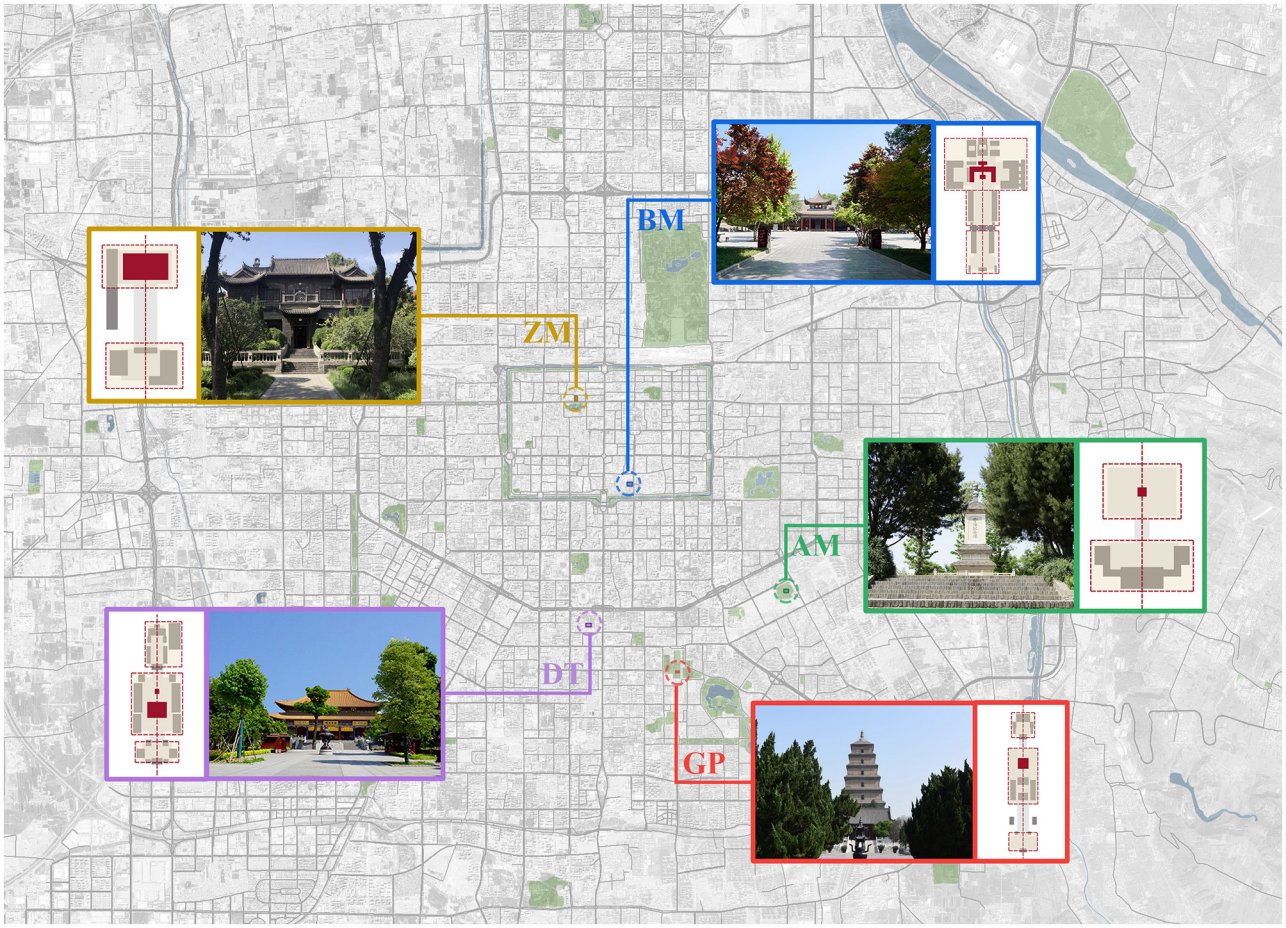
Location of the cases.

We chose Xi'an City as the site for our experiments. Xi'an with numerous historical and cultural sites is a typical representative of state-list 100 famous historical and cultural cities in China. Study of Xi'an may give reference for other historical and cultural cities around the world. Specifically, we selected five sites (i.e., the Greater Wild Goose Pagoda [GP], Beilin Museum [BM], Airsea Monument [AM], Daxingshan Temple [DT], and Zhiyuan Memorial Hall [ZM]) for the case group, as shown in Figure 1, which rely on the profound cultural connotation of historical accumulation and their site and spirit preserved today. They also act as the most famous cultural heritage sites in Xi'an. The sites consist of yards, squares, trees, and historical buildings, with an axis sequence, and are the most common types of cultural heritage sites in China.

We took colored photographs of each case in spring and then selected three pictures with a similar angle and composition for each case. For the control group, we removed the historical building in each case using Photoshop. Next, we selected a high-level reference for the control group and conducted a comparison to verify which group obtained better results in terms of restoration. We ensured that the case and control groups corresponded with each other.

We invited more than 700 students from Xi'an University of Architecture and Technology to participate in our experiments; 732 of whom completed the questionnaire and 68 of whom participated in the physiological experiments. We randomly assigned the students into five case groups.

### 2.2. Measures

A questionnaire and a physiological experiment are both used in this study. The questionnaire is used to reflect participants' feelings via a self-report approach, while a physiological experiment could show one's body responds to a certain environment via electrical signal devices. Either one of them is lacking in comprehensiveness or objectivity. The assessment method that simultaneously monitors the mental and physiological states of the participants could better present people's perceptions of the environment.

#### 2.2.1. Questionnaire

We adopted a questionnaire on WJX (a web questionnaire site) composed of three parts. The first part of the questionnaire consisted of three general questions about the participant's age, gender, and familiarity with the case city. The second part was the Perceived Restorative Scale (PRS) in the Chinese version (Ye et al., 2010), which used a 7-point scale comprising 22 items for the dimensions of being away, compatibility, fascination, and extent. The last part of the questionnaire included three questions on the participants' familiarity with and preference for the case. The main design variables of the questionnaire included the participants' PRS score, the score for the four dimensions, preference, and familiarity.

#### 2.2.2. Physiological experiment

We conducted two types of experiments, including an electrophysiology study and an eye movement test, to determine the physiological responses related to restoration. The experiments involved several indices, such as skin conductance response (SCR) and heart rate variability (HRV), in the electrophysiology study.

Scholars generally use HRV and SCR as measures of restoration. In this study, we used two types of wireless wearable devices with transmitters. The first one was a photoplethysmography device for HRV. We connected a short wire to the participants' ears to detect heart rate signals. For this test, we used two dimensions, that is, the time domain and frequency domain, and five variables, namely, MeanIBI, RMSSD, PNN50, SDNN, and LF/HF. The MeanIBI variable is an RR interval representing the time interval of R wave peaks in two adjacent heartbeats. The RMSSD variable represents the root mean square value of the difference between the adjacent RR intervals, and the SDNN variable represents sympathetic nerve activity, with ms as the unit. In addition, the PNN50 variable represents the ratio of the number of adjacent RR interval differences >50 ms to the total number of RR intervals, with % as the unit. The frequency–domain indicator LF/HF represents the balance between the sympathetic and parasympathetic nerves.

The second device was for the electrodermal activity (EDA) for the SCR. We connected two short wires to the pad of the index and middle fingers of the participants to obtain the SCR signals. We set two variables for this measure: SC and tonic. The former represents the skin conductance, whereas the latter is a gradient signal and its unit is μS.

The eye movement test is a procedure for monitoring the eye movement and gaze direction of a subject when looking at a specific target through eye-tracking technology and for performing correlation analysis. We used an eye movement tracking system (Sweden Tobii, TX300) for real-time synchronous recording. For this measure, we selected three eye movement indices, including the mean count of eye blinks, the total duration of all fixations, and the fixation position.

Finally, we used the ErgoLAB Human–Machine–Environment cloud platform to realize the real-time and synchronous acquisition of human–machine–environment multidimensional data, such as eye-tracking and physiological data. We also conducted data processing and analysis on the ErgoLAB multimodal data synchronization cloud platform.

### 2.3. Procedures

#### 2.3.1. Pilot study

At the beginning of the survey, we showed a QR code from WJX (a web questionnaire site) on a projector screen and asked each participant to scan the code to access the questionnaire. The questionnaire included questions about the participant's age, gender, and familiarity with the case city. Next, we displayed a group of pictures of a cultural heritage site (or from the control group) and instructed the participants to rate the restorative value of the site on the Chinese version of the PRS. We projected each picture on a screen for 10 s before changing it to another picture. When the participants finished answering the PRS, we displayed the corresponding pictures from the control group (or cultural heritage sites) and instructed them to complete another PRS. We used three additional items to measure the participants' familiarity with and preference for the cases. The participants spent ~10 min to answer the questionnaire.

#### 2.3.2. Quasi-experimental design

**Figure 2:**
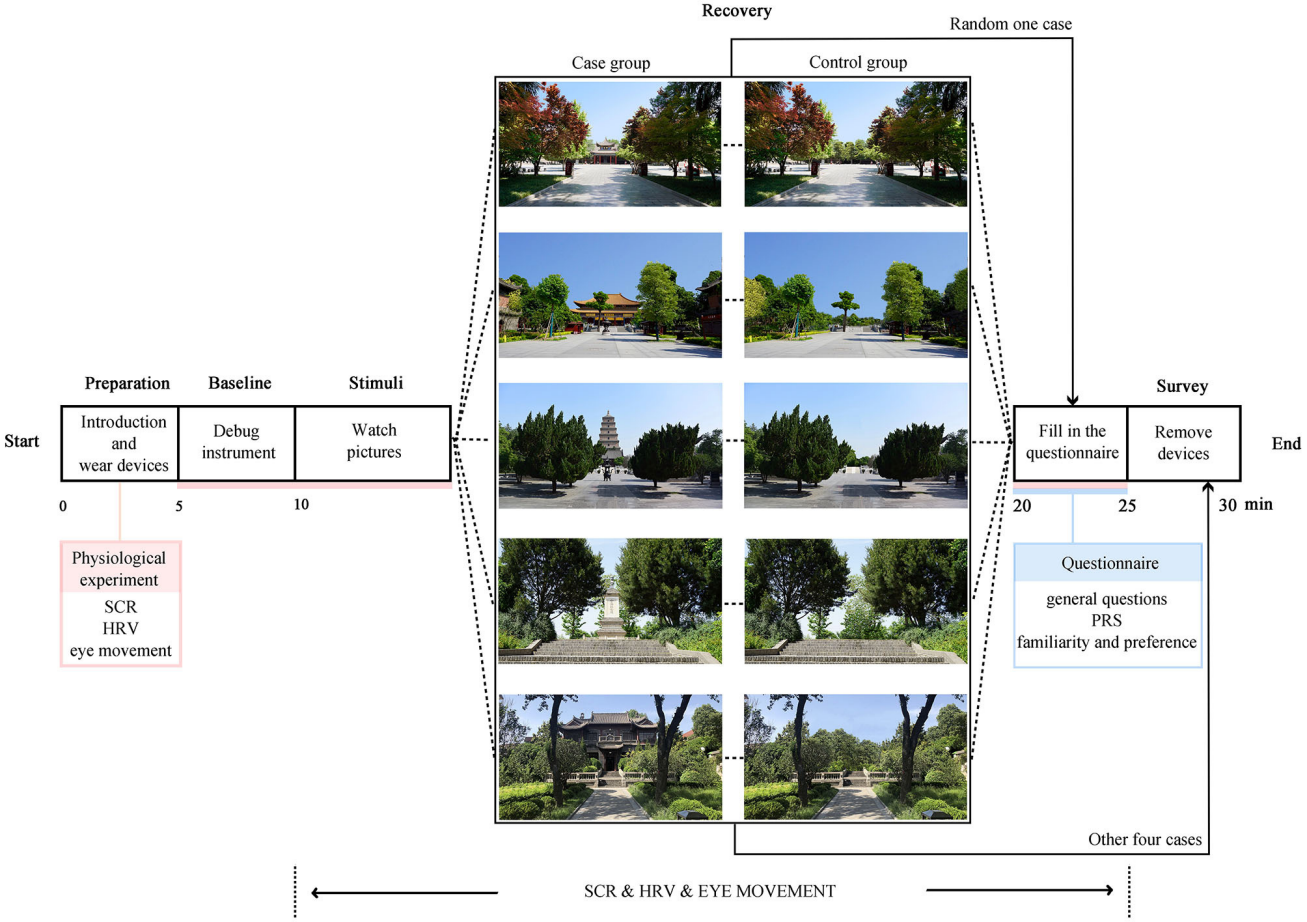
The procedure of the quasi-experiment.

The procedure is presented in Figure 2. First, the participants equipped themselves with the wearable devices we calibrated for the electrophysiology study and eye movement test. Second, the participants sat in front of a projector screen to view the pictures of one cultural heritage site (or pictures from the control group). We projected each picture on the screen for 10 s before showing the next one and then instructed the participants to complete the PRS after the activity. Third, we showed the participants the corresponding control group pictures (or pictures of one cultural heritage site), projected in the same way, and asked them to complete the PRS once again after the activity. Finally, three additional items were used to measure the participants' familiarity with and preference for the sites. After the completion of the abovementioned items, the participants can remove the relevant equipment. Specifically, we showed the participants pictures of other cultural heritage sites and the corresponding pictures from the control group. The duration of the experiment was ~30 min.

## 3. Results

### 3.1. PRS score in the pilot study

**Figure 3:**
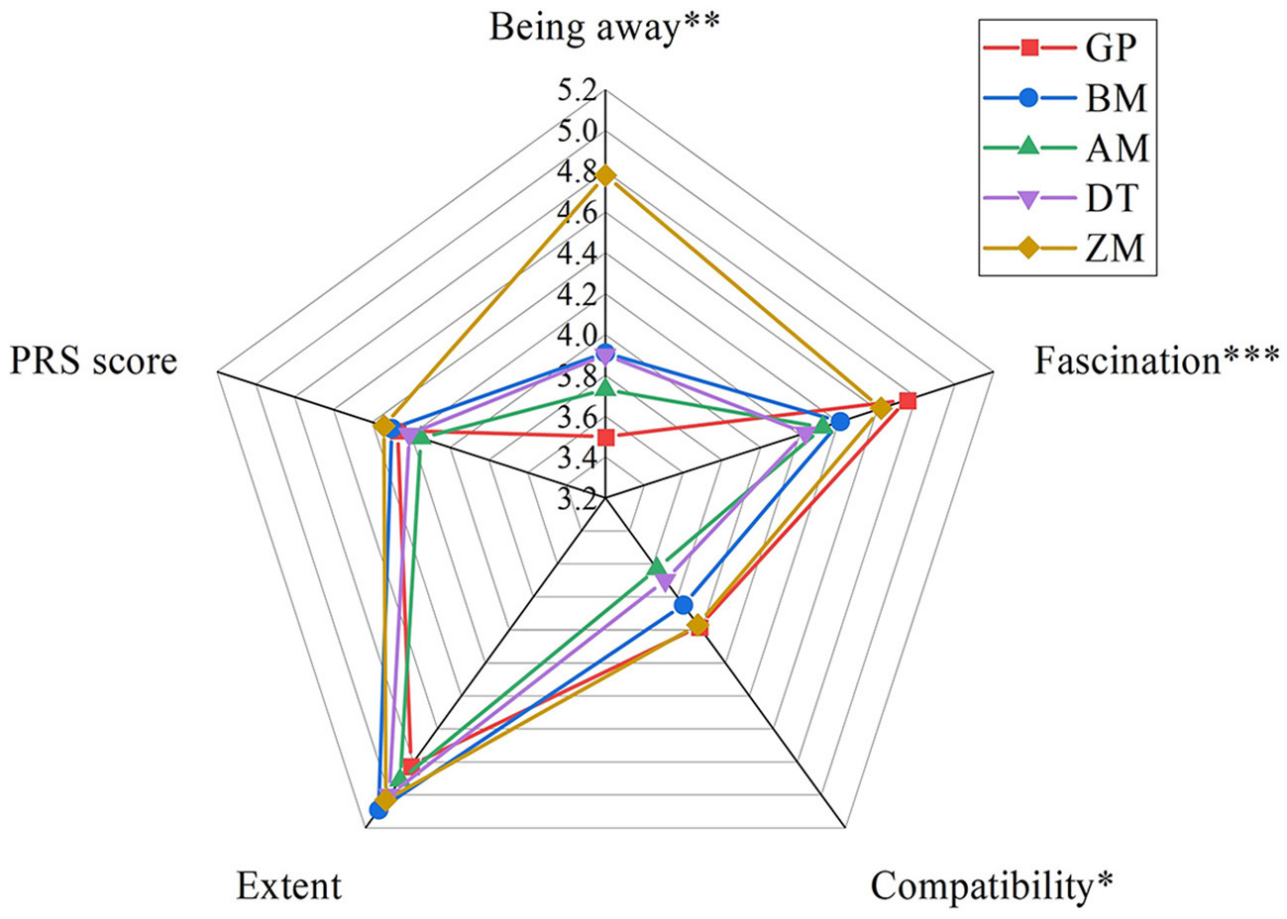
PRS score of the cases. Statistically different: ^***^P ≤ 0.001; ^**^P ≤ 0.01; ^*^P ≤ 0.05.

In terms of valid questionnaires, we collected 156 for GP, 145 for BM, 149 for AM, 154 for DT, and 128 for ZM. The PRS scores indicated that the sites had a high restorative value. The mean of the PRS scores ranged from 4.15 to 4.34, which was fairly high on a 7-point scale. ZM had the highest mean PRS score and mean score in the being away dimension, whereas AM received the lowest score in the being away dimension. Meanwhile, GP received the highest score in the fascination dimension, as shown in Figure 3.

The statistical difference analysis results of the dimensions of being away, fascination, and compatibility fully represented the special characteristics of GP and ZM. Specifically, GP showed a significant difference in the being away, fascination, and compatibility dimensions, whereas ZM exhibited a significant difference in the fascination and compatibility dimensions, especially between AM, DT, and ZM, as shown in Table 1.

**Table 1:** Statistical difference between being away, fascination, and compatibility.

**Cases**	**Being away**	**Fascination**	**Compatibility**
GP-BM	0.42^**^	0.34^**^	0.13
GP-AM	0.23	0.43^**^	0.35^*^
GP-DT	0.40^**^	0.53^***^	0.28^*^
GP-ZM	0.28^*^	0.13	0.01
AM-ZM	0.05	0.30^*^	0.34^*^
DT-ZM	0.13	0.39^**^	0.27

Statistical difference: ^***^P ≤ 0.001; ^**^P ≤ 0.01; and ^*^P ≤ 0.05.

Comparison of the mean value of being away, fascination, and compatibility of PRS score for five cases.

We collected a total of 732 valid questionnaires for the case and control groups. The results of the comparative study between the cultural heritage sites (case group) and corresponding green spaces (control group) in Table 2 show that the overall PRS score of the case group was higher. In addition, the scores of three dimensions, namely, being away, fascination, and extent, were higher in the case group than in the control group, especially fascination. However, the standard deviation of fascination, compatibility, and extent was lower in the case group than in the control group. This finding implied that the scores of the case group were more concentrated than those of the control group and indirectly indicated that the score of the fascination dimension was higher than that of the other dimensions. The score and standard deviation of the compatibility dimension were also higher in the control group than in the case group, which demonstrated a higher but more dispersed distribution and indirectly illustrated that the high score was not absolute. The results emphasized the significant difference in the fascination dimension between the cultural heritage sites (case group) and their corresponding green spaces (control group).

**Table 2:** Independent sample *t*-test determining the difference between the two groups.

	**Case group**			**Control group**		
	**Mean**	**SD**	**SE**	**Mean**	**SD**	**SE**
Being away	3.76	1.18	0.04	3.74	1.16	0.04
Fascination^***^	4.46	1.24	0.05	3.99	1.30	0.05
Compatibility	3.82	1.25	0.05	3.85	1.32	0.05
Extent	4.97	1.22	0.05	4.88	1.27	0.05
PRS score	4.25	–	–	4.12	–	–

Statistical difference: ^***^P ≤ 0.001.

Comparison of the mean value of the PRS score for the two groups.

### 3.2. PRS score in the quasi-experiment

**Figure 4:**
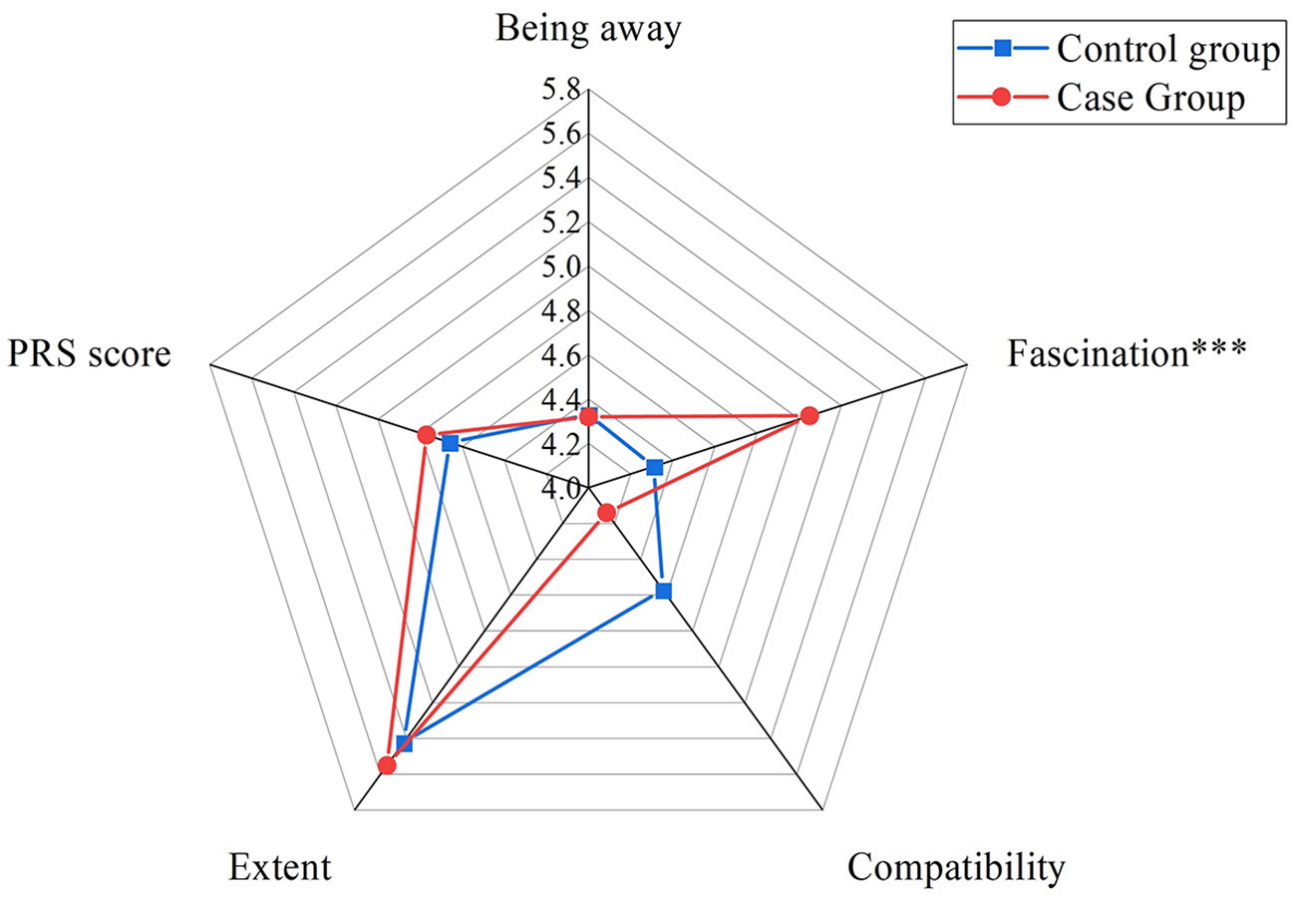
Comparison of PRS score between the two groups. Statistical difference: ***P ≤ 0.001.

We collected 63 valid questionnaires for the case group and another 63 for the control group. The results of the PRS comparison were similar to those of the pilot study. The PRS and fascination and extent dimension scores were higher in the case group than in the control group, which reflected high restorative value, as illustrated in Figure 4.

**Figure 5:**
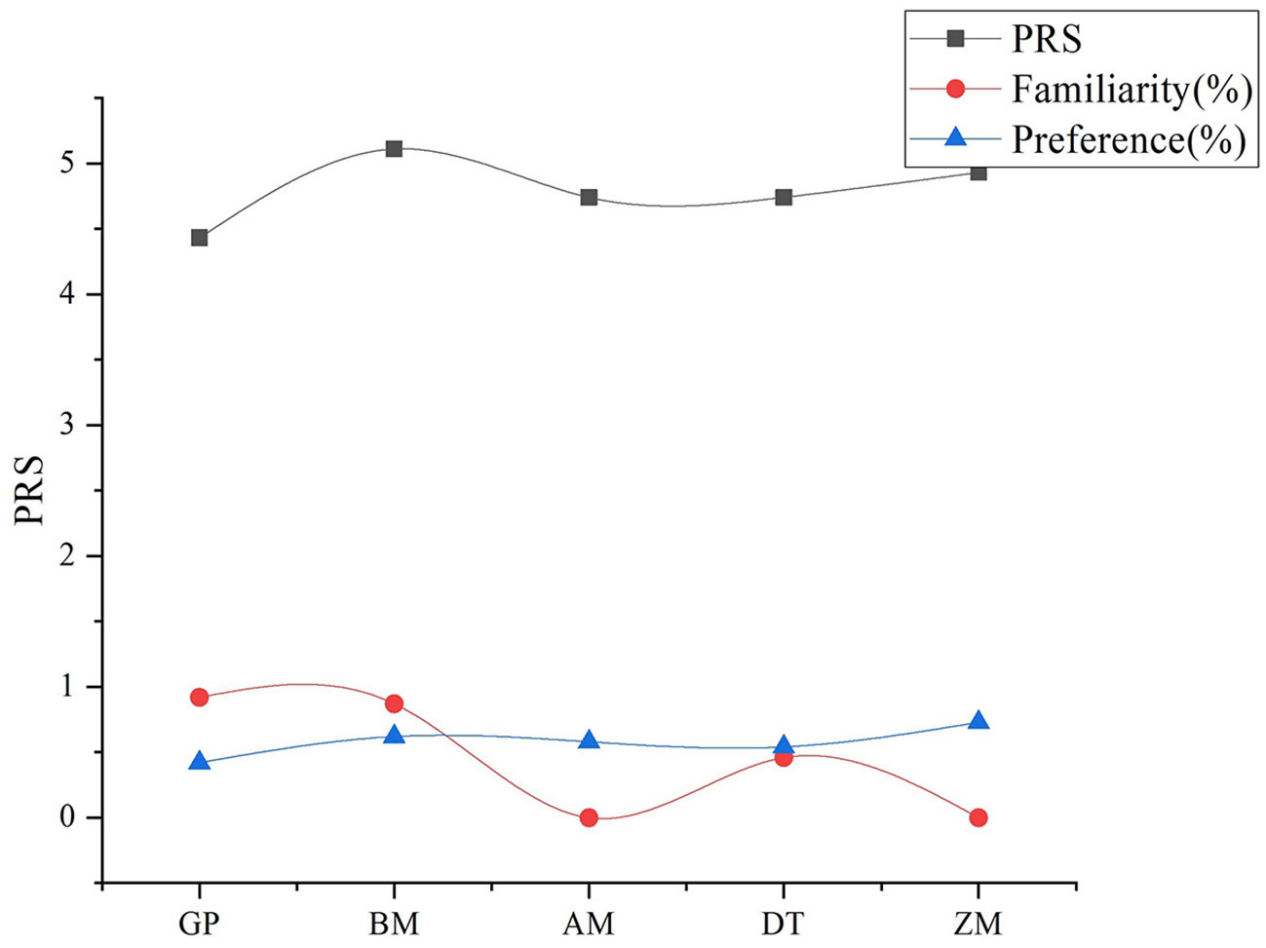
PRS score, familiarity, and preference of the cases.

From Figure 5, we can see the relationship between the PRS score and familiarity and preference. Specifically, GP received the highest familiarity but lowest PRS score, whereas ZM and BM demonstrated the highest preference as well as the highest and the second-highest PRS scores, respectively. These results implied that low familiarity but high preference led to a high PRS score, and preference exerted a considerable influence.

### 3.3. Physiological experiment data analysis

#### 3.3.1. SCR, HRV, and eye movement analysis of different cases

Table 3 lists the SCR, HRV, and eye state index results of different cases. BM demonstrated the lowest SC and tonic values, which implied the highest relaxation level, whereas GP exhibited the highest SC and tonic values, which may be attributed to its ability to induce excitement and restorative effects. The HRV index results revealed that nearly all the data were long, which implied relaxation. Meanwhile, ZM and BM showed the lowest and highest SDNN values, respectively, whereas AM and ZM demonstrated the highest and lowest LF/HF values, respectively. In addition, the MeanIBI and RMSSD outcomes of BM were the longest. In our experiments, we projected each picture on the screen for 10 s, and the eye state results indicated that the eye blink count was few (once every 2 s or longer), and the total fixation duration was more than 7 s. This finding implied a relaxed eye state. Among all the cases, AM exhibited the lowest blink count, whereas BM showcased the longest fixation duration.

**Table 3:** SCR, HRV, and eye state index of the cases.

**Type**	**Index**	**GP**	**BM**	**AM**	**DT**	**ZM**
SCR	SC (μS)	3.83	3.61	3.83	3.82	3.79
	Tonic (μS)	3.58	3.35	3.54	3.54	3.55
HRV—time domain	MeanIBI (ms)	795.14	792.57	789.43	795.91	791.66
	RMSSD (ms)	96.19	104.90	90.99	88.66	89.05
	PNN50 (%)	0.24	0.22	0.25	0.22	0.21
	SDNN (ms)	78.35	81.48	73.46	73.04	69.41
HRV—frequency domain	LF/HF	0.62	0.56	0.68	0.62	0.54
Eye movement	Blink count (*N*)	5.21	4.56	4.24	4.75	4.58
	Total fixation (s)	7.19	7.50	7.50	7.02	7.48

This table shows the difference between different cases.

Furthermore, AM, DT, and ZM were the special cases, as shown in Table 4. The difference in the LF/HF values between AM and ZM was significant, and the blink count comparison was also significant between GP and AM. The total fixation duration for DT also differed from that for BM, AM, and ZM.

**Table 4:** Statistical difference in the physiological index of the cases.

**Cases**	**Index**	**Difference in mean value**
AM-ZM	LF/HF	0.14^*^
GP-AM	Blink count	0.97^*^
BM-DT	Total fixation	0.47^*^
AM-DT	Total fixation	0.47^*^
ZM-DT	Total fixation	0.46^*^

Statistical difference: ^*^P ≤ 0.05.

Comparison of the mean value of LF/HF, blink number, and total fixation for the five cases.

#### 3.3.2. SCR, HRV, and eye movement data comparison analysis of the two groups

**Figure 6:**
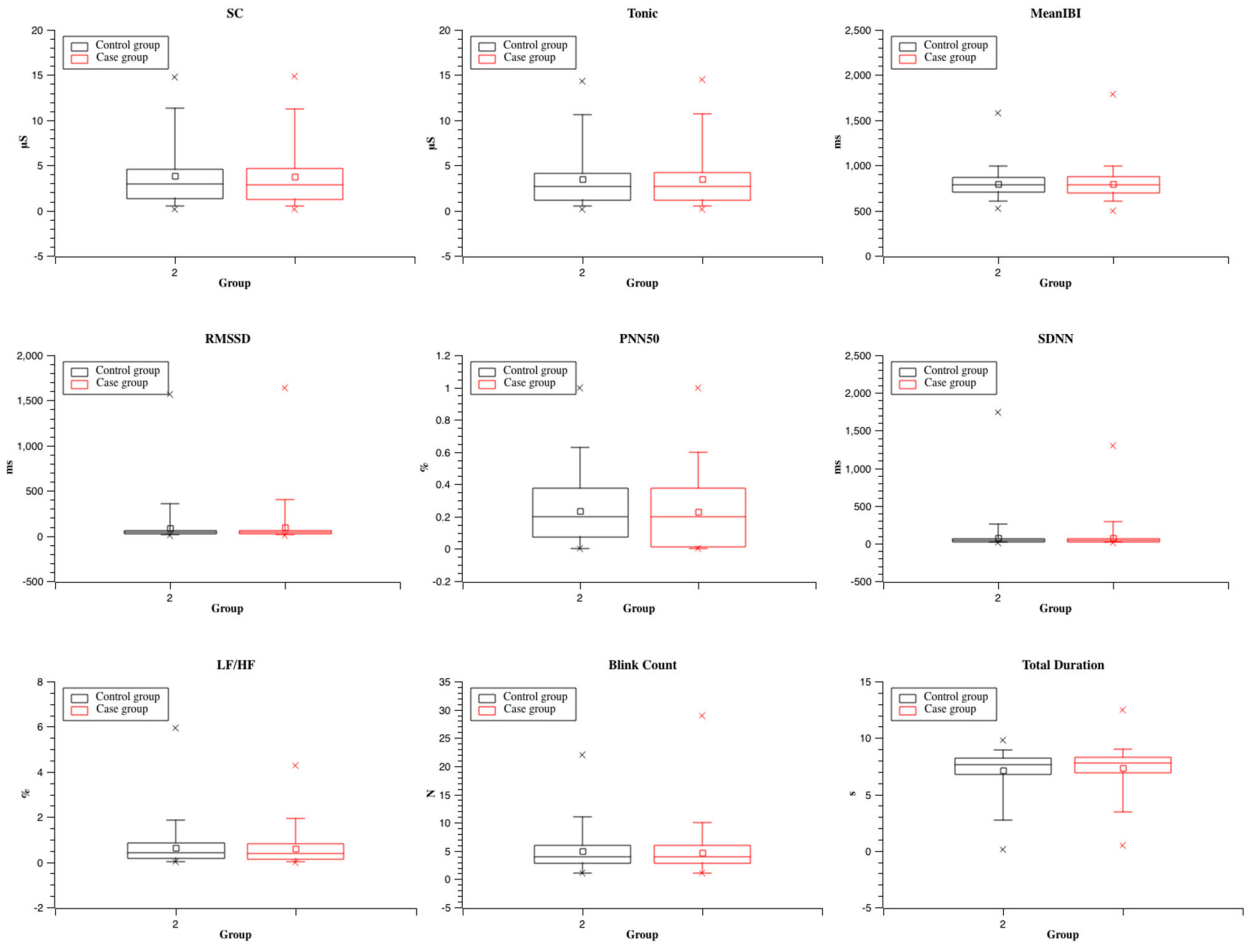
Comparison of SCR, HRV, and eye state between the two groups.

The SCR comparison in Figure 6 shows that the mean value of SC and tonic was lower in the case group than in the control group. This finding indicated that the case group promoted restoration and relaxation among the participants. The HRV results showed that the mean value of MeanIBI and RMSSD was long, but the PNN50 value was lower in the case group than in the control group. Similarly, this finding implied that the case group had a relaxing effect on the participants. The SDNN results showed that physiological arousal was higher in the case group than in the control group, indicating higher excitement based on the abovementioned index. Moreover, the LF/HF value was lower in the case group than in the control group, emphasizing that the cultural heritage site was attractive to the participants. The eye state results revealed that the mean blink count was low, and the fixation duration was longer in the case group than in the control group. This finding indicated that the case group relaxed the participants' eyes and attracted their interest.

The two groups also differed in terms of visual perception, as shown in Table 5. The areas in red indicated a long fixation time, whereas those in green showed a short fixation time. The results of the fixation heat map comparison revealed that the participants fixated and concentrated on the historical buildings in the case group. We also observed accumulation, but fixation in the control group was highly dispersive. This finding demonstrated the participants' high level of attraction to the case group, especially the restorative fascination dimension.

**Table 5:** Fixation heat map pictures of the two groups.

**Cases**	**Case group**	**Control group**
GP	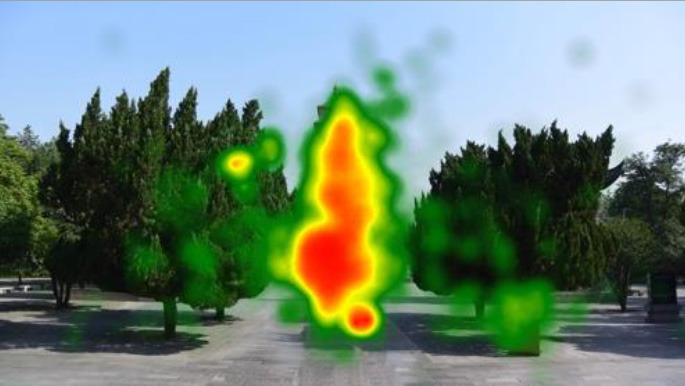	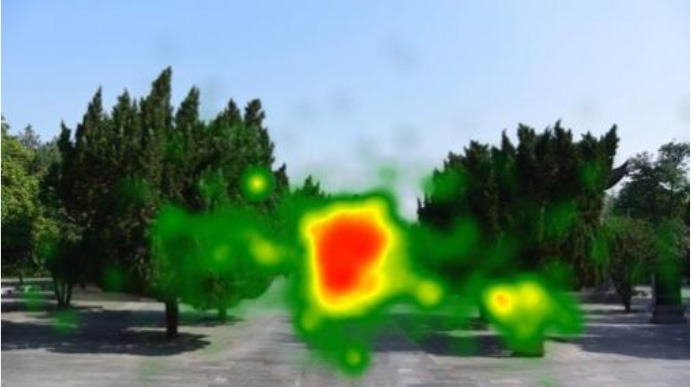
BM	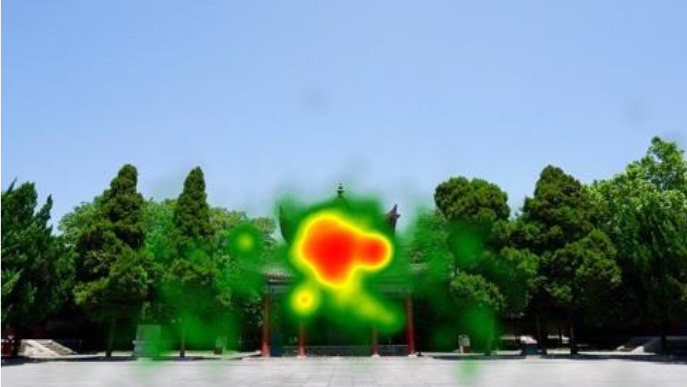	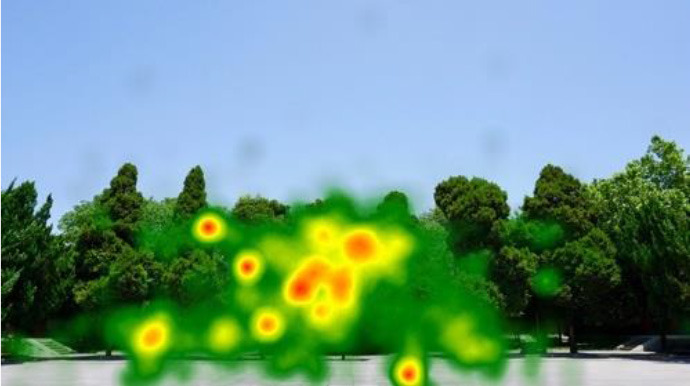
AM	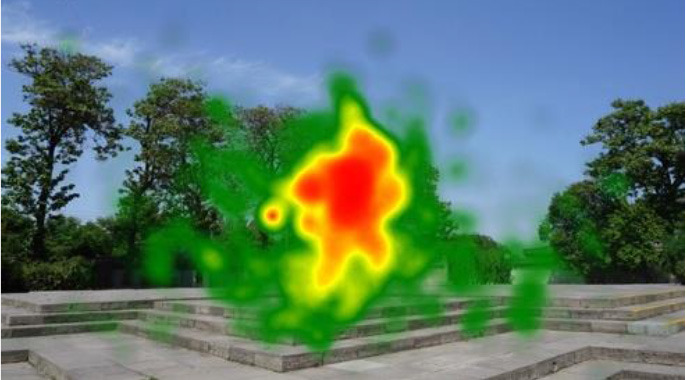	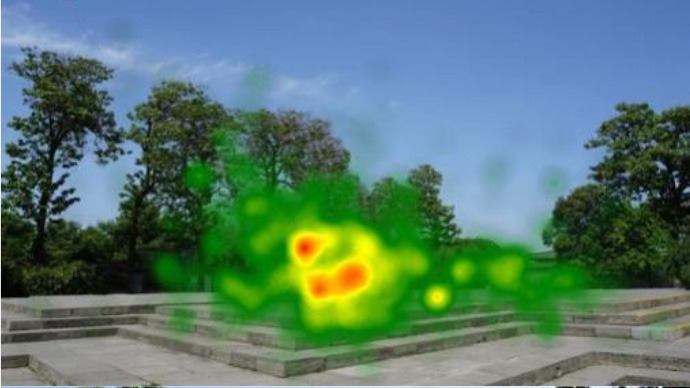
DP	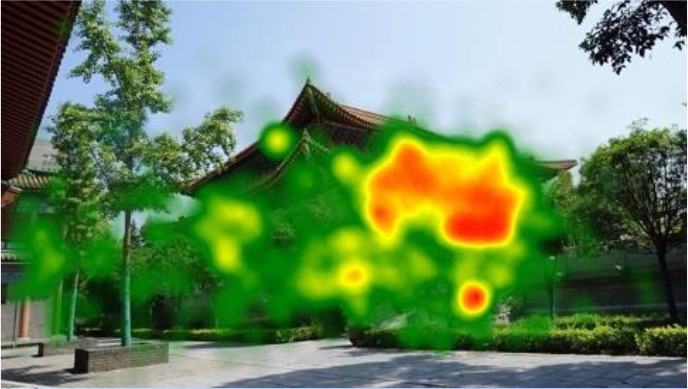	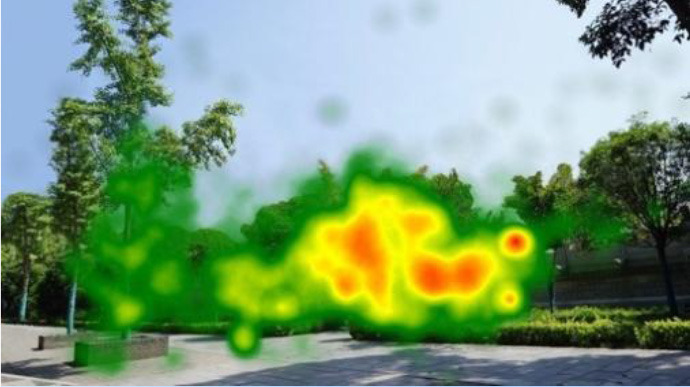
ZM	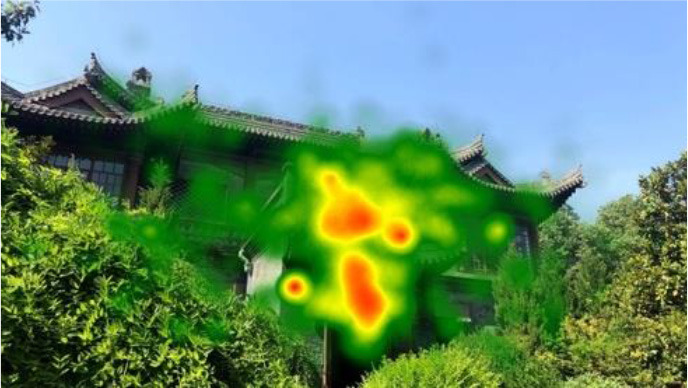	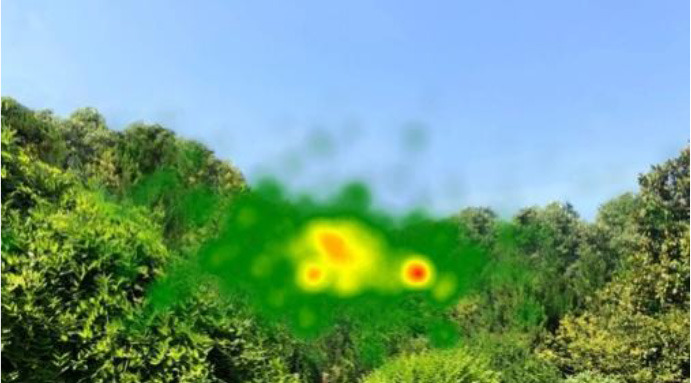

## 4. Discussion

In this study, we find a new application of the high restorative effects of cultural heritage sites (Fornara and Troffa, 2009; Scopelliti et al., 2019) by using various assessments in the Chinese context. The PRS, SCR, HRV, and eye movement data indicate the high restorative effects and considerable attraction value of the cultural heritage sites. We obtain several results from the experiments. First, the restorative effects of the cultural heritage sites are similar to or higher than those of the green spaces. Second, the cultural heritage sites demonstrate restorative effects and attraction value, which are different from the green spaces. Third, the reason behind this difference is that historical buildings with substantial esthetic value are attractive. Finally, the size, height, and esthetic value of the historical buildings influence the restorative and attraction value of the cultural heritage sites, which may induce excitement when the historical buildings are magnificent.

By comparing the two groups, we find that the cultural heritage sites prevail over the green spaces, which are similar in composition but different in terms of cultural characteristics, such as the presence of historical buildings. The data indicate that cultural heritage sites have stronger restorative effects and higher attraction value than green spaces. Moreover, the experiment results show the high relevance and restorative value of the historical sites, especially in terms of the fascination dimension (Scopelliti et al., 2019). In addition, the case group exhibits a lower SC value and longer MeanIBI than the control group (Ulrich et al., 1991; Wang et al., 2016; Stigsdotter et al., 2017; Yin et al., 2020), but the change degree of the SCR and HRV indices for the two groups is fair. Eye movement can reveal a relaxed state (Marek et al., 2018), and a low blink count and long fixation time indicate a restored state from fatigue and attraction to attention (Ren et al., 2019). Furthermore, the fixation heat map presents the participants' attraction to the cultural heritage sites. Previous studies proposed that eye movement in image perception can reveal attentional and cognitive processes (Holmqvist et al., 2011), and long and focused fixations indicate considerable interest in and preference for the fixation point (Nummenmaa et al., 2006). The results indirectly prove that the restorative and attraction value of the cultural heritage sites is higher than that of the green spaces. This finding provides a new resource for mental health that may be useful in urban planning, design, and policymaking. That is, cultural heritage may help in the construction of cities that are conducive to mental health to a certain extent.

The contribution of our research is our findings on the effect of historical buildings, which can raise PRS scores, especially in terms of the fascination dimension; reduce the SC level; and extend the MeanIBI outcome. Historical buildings may also induce excitement, which is reflected in the SDNN index. The fixation position and duration and blink count for the historical buildings are also significantly different. This finding indicates that the attractiveness of cultural heritage sites has restorative effects and can help explain the significance of cultural heritage historical buildings to restoration. The significance of historical buildings may be similar to that of natural and other types of elements (Wang et al., 2016), but they are different. This finding highlights the importance of the conservation and design of historical buildings as well as their value and shows that cultural heritage has not only conservation value but also esthetic value, which may have restorative effects and attraction value.

Familiarity and preference can also influence the restorative effects of cultural heritage sites. Low familiarity but high preference can lead to a high PRS score, and preference exerts a substantial influence. This finding is consistent with that of previous studies (Weber and Trojan, 2018) and implies differences in the influence of preference. Some scholars argued the importance of historical monuments and attractions in addition to specific environmental features appreciated by the public (Van Berkel et al., 2018).

The comparison results of the five cases demonstrate similarities and differences, including in some of the special cases. Nearly all the cases exhibit satisfactory restorative effects through the different data. ZM received the highest mean PRS and being away dimension scores in the pilot study, whereas GP received the highest score in the fascination dimension, and BM received the highest score in the extent dimension. In the EDA, BM demonstrates the lowest SC and tonic values, whereas GP and AM exhibit the highest SC value. For the HRV index, BM presents the longest MeanIBI and RMSSD outcomes and the highest SDNN value. In line with our expectations, BM provides the best restorative effects while being the most attractive among the five cases. GP is the most special case, as it received the highest score in the fascination dimension. However, the results of the different types of data are contradictory. We infer that GP induces excitement and restorative effects simultaneously. This finding implies that policymakers and urban management authorities can utilize cultural heritage elements for different mental health purposes. Urban planners may use tranquil cultural heritage sites with small and exquisite buildings for restoration, as magnificent buildings with high attraction value may induce excitement and restorative effects.

In our study, we also use a different connection and combination of electrophysiology response and eye movement examinations. The connection may explain the difference between the two similar groups and help in finding tiny cues and provide an appropriate approach for examining a city's various environments. Some of the skillfully constructed environments may not have a long HRV outcome and low SCR value, because their attractiveness may lead to excitement. However, they may also have considerable restorative effects, as indicated by their PRS score and eye movement measurement results. Furthermore, the eye movement data can explain why some of the sites have restorative effects whereas others have restorative effects with attraction value. The abovementioned results can provide data support for the restoration research of cultural heritage.

This study opens up other research possibilities for cultural heritage. Further studies may analyze the mental restoration mechanism of cultural heritage sites and their relationship with the space characteristics of their location. Thus, the value of other cultural heritage sites is worth exploring.

## 5. Conclusion

In this study, we identify the significant mental restorative effects of cultural heritage sites and employ multiple evaluation indices to assess and prove their restorative effects and attraction value. Among the different dimensions, fascination is the most significant. The findings of this study have theoretical and practical significance. Specifically, the findings fill a significant knowledge gap and provide concrete evidence that cultural heritage sites are mentally restorative and different from green spaces. Moreover, cultural heritage sites are special in terms of the fascination dimension, which may be attributed to the presence of historical buildings to a certain extent. This study may help the government and the public notice and focus on the mental health effects of cultural heritage and provide enlightenment on the construction and conservation of and policymaking for cultural heritage sites.

## Data availability statement

The raw data supporting the conclusions of this article will be made available by the authors, without undue reservation.

## Ethics statement

Ethical review and approval was not required for the study on human participants in accordance with the local legislation and institutional requirements. Written informed consent from the patients/participants or patients/participants legal guardian/next of kin was not required to participate in this study in accordance with the national legislation and the institutional requirements.

## Author contributions

SW: conceptualization, methodology, writing—reviewing and editing, and funding acquisition. YX: formal analysis, data curation, writing—original draft preparation, and project administration. XY: investigation, resources, and software. YZ: methodology, validation, and supervision. PY: data curation, visualization, and investigation. YJ: resources and visualization. KW: investigation. All authors contributed to the article and approved the submitted version.
